# Management of Wet Age-Related Macular Degeneration in Spain: Challenges for Treat and Extend Implementation in Routine Clinical Practice

**DOI:** 10.1155/2019/9821509

**Published:** 2019-09-30

**Authors:** A. García-Layana, J. García-Arumí, M. S. Figueroa, L. Arias Barquet, J. M. Ruíz-Moreno, L. Monclús-Arbona, The Spanish AMD Multicenter Group

**Affiliations:** ^1^Clínica Universidad de Navarra, Avenida de Pío XII 36, 31008 Pamplona, Spain; ^2^Hospital de la Vall d'Hebrón, Passeig de la Vall d'Hebrón 119-129, 08035 Barcelona, Spain; ^3^Vissum Madrid, Santa Hortensia 58, 28002 Madrid, Spain; ^4^Hospital de Bellvitge, C/ Feixa Llarga s/n, L'Hospitalet de Llobregat, 08907 Barcelona, Spain; ^5^Hospital Universitario Puerta de Hierro-Majadahonda, Joaquín Rodrigo, 2, 28222 Madrid, Spain; ^6^Bayer Hispania, S.L., Av. Baix Llobregat, 3-5, 08970 Sant Joan Despí, Barcelona, Spain; ^7^IQVIA Information, Barcelona, Spain

## Abstract

**Purpose:**

To ascertain wet AMD (wAMD) management patterns in Spain.

**Methods:**

A two-round Delphi study conducted through a questionnaire-based survey designed from literature review and validated by an independent Steering Committee.

**Results:**

Forty-nine retina specialists experienced in wAMD participated by answering the two-round study questionnaire. Retina specialists are the main responsible for wAMD diagnosis and monitoring, including visits and associated procedures, with a median time per visit of 15 minutes. Standard treatment strategies are based on anti-VEGF administration, including standard loading dose administration followed by maintenance with aflibercept or ranibizumab (81% of patients). Although treat and extend (T&E) dosing strategy is considered as optimal for wAMD management (78% of the panelists), the main routine healthcare limitations (i.e., visits overload, reduced staff, short visit time, coordination issues, lack of facilities) conduct to self-defined “flexible” strategies, based on T&E and pro-re-nata (PRN) protocols.

**Conclusion:**

Proactive treatment patterns (T&E) are the preferred ones by the retina specialists in Spain. However, their proper implementation is difficult due to healthcare resource limitations, as well as organisation and logistic issues. The use of anti-VEGF agents with longer duration of action could facilitate the use of strict T&E approaches according to routine clinical practices.

## 1. Introduction

Age-related macular degeneration (AMD) is a chronic, progressive, and severe disease of the central retina and is the main cause of irreversible blindness in the Western world and Asia-Pacific countries [1, 2]. Up to date, 8.7% of the worldwide population has AMD [3]. The wet form of the disease is responsible for more than 90% of the severe central visual acuity (VA) loss associated with AMD [4].

Wet AMD (wAMD) has no cure even though an appropriated treatment could delay disease progression, avoiding the negative impact of vision loss in the quality of life of these patients [5, 6]. According to the most recent AMD guidelines [7, 8], the intravitreal administration of antivascular endothelial growth factor agents (anti-VEGF) constitutes the standard of care for wAMD patients due to the promising results provided by these therapies during clinical development [9].

However, the success of anti-VEGF therapy is closely linked to strict intravitreal treatment patterns [10] that seem not to be properly addressed in routine clinical practice [11, 12], conducting to poorer outcome results for wAMD patients than the expected according to clinical trials [13, 14]. During the first year of treatment, results in real world practice seem to be closer to those reported for each drug [11, 12]. However, during the second year, treatment dosing increases in “flexibility” due to the rise in the amount of patients, stress of monthly assessments, and economic burden, having a negative impact on outcomes and becoming an especially relevant issue [11, 12].

Healthcare limitations are a reality in routine clinical practice. However, strict management protocols should be considered mandatory to guarantee optimal results for wAMD patients, involving drug selection and dosing strategy [15]. In order to choose the most appropriate therapeutic approach, it is necessary to consider several factors including patient profile, disease characteristics, drug access, healthcare resources available, management protocols, and healthcare burden, among others [14, 16]. In this regard, a Delphi study was conducted to describe wAMD management in Spain based on anti-VEGF administration. Challenges, limitations, and preferences of the retina specialists were assessed to identify the most appropriate treatment and dosing strategies for these patients in routine clinical practice.

## 2. Methods

### 2.1. Study Design and Development

According to the study objectives, a two-round Delphi methodology [16–18] was considered as the most appropriate due to its capability to define answer consensus and ratification. An ad hoc study questionnaire was developed after an exhaustive literature search on wAMD management and anti-VEGF evidence, being analysed and validated by a Steering Committee conformed by 5 ophthalmologists with retina expertise, from 5 different Spanish centres. The questionnaire included a final set of 38 questions, comprising 4 main sections: (i) participant profile, (ii) general management of wAMD, (iii) wAMD diagnosis, and (iv) wAMD treatment. Treatment strategy options were categorized as “strict” or “flexible” according to the agreement level with the recommended patterns [7, 8] and product technical conditions [10]. Thus, strict pro-re-nata (PRN) would involve monthly monitoring and immediate on-demand treatment after loading dose, while strict treat and extend (T&E) would involve regular preplanned treatment administration after loading dose administration, including progressive increase in the injection periods. Flexible strategies were considered as deviations of the standard strategies, mainly represented by an irregular monitoring and/or treatment of the patients and attributable to routine clinical practice needs.

The study questionnaire was designed to be filled online by 50 retina specialists selected according to the following criteria: (i) ≥5 years in wAMD management in Spain and (ii) to be the current responsible for wAMD management in the centre (including treatment administration and follow-up). Main contact for study recruitment was conducted by email, and the interested participants signed an agreement, committing to answer both Delphi rounds (approximately 20 minutes each). The access link to the questionnaire was emailed to each participant, together with a personal access individually provided in a separated email. All questions were designed to be answered considering the experience of each panelist according to their routine clinical practice. Questions focused on treatment strategy prioritization were rated by the panelists, and each rank level was converted into scoring punctuation, providing mean priority scores per option.

In Spain, there are more than 300 hospitals with ophthalmology service and more than 600 retina specialists experienced in the management of wAMD. Since the sample size for a Delphi panel depends on study objectives, available resources, and panel nature (ranging from 10–15 for homogeneous groups to several hundreds in heterogeneous groups [19]), a sample of 50 retina specialists (a homogeneous group of healthcare professionals with different realities) was considered as enough for this study purpose, prioritizing those from the biggest hospitals of the different Spanish regions. In addition, all participants had been previously involved in wAMD and Delphi studies in Spain, being experienced not only in the disease, but in methodology [16].

The same study questionnaire was used in both rounds, with minor adaptations, so that answers could be reviewed, rechecked, and confirmed, providing robustness to the final study results. The first Delphi round was carried out in April 2017, and the second in June 2017. The results collected in the first round were discussed by the Steering Committee, and some items were slightly reworded for clarification. Aggregated results were provided to the panelists before the second Delphi round, where the empty questionnaire was resent to the participants to be responded (none of the participants had access to their previous individual answers). Consensus was predefined as homogeneity or consistency of opinions among experts and understood as a reduction of answer dispersion between data provided in the first and second Delphi rounds.

### 2.2. Result Analysis

A descriptive analysis was conducted per outcome, providing number of responses (*n*) and percentage (%), mean result, and standard deviation (SD). For abnormal outcomes, median and confidence interval were calculated. Results of the second round were used as the final validated data of the study. Considering the nature of the questions, as well as the different scoring systems used in the study, consensus was reached with ≥60% of agreement per item. Additionally, the dispersion in the answers was also considered, being considerably lower than in first-round responses.

## 3. Results

### 3.1. Participant Profile

A total of 49 retina specialists from all around Spain participated in both Delphi rounds. Mean (SD) age of participants was 49 (7) years, and they accounted for a mean (SD) of 16 (6) years of experience in the wAMD patients' management. Most participants (82%) were frequently involved in ophthalmology clinical trials (mean (SD) 3.7 (2.7) trials per year).

### 3.2. Burden of wAMD Management in the Ophthalmology Services

wAMD management represents 25% (18) of the ophthalmology visits in Spain, with a median time (P25–P75) per visit of 15 minutes (10–15 minutes). This time considers the average time per patient including treatment administration and other procedures, when needed, and excluding patient waiting time.

wAMD patients are usually referred to the retina specialist by other medical doctors (40%), from the same centre or related (i.e., ophthalmologists from primary care), as well as from the emergency units (32%). The retina specialist is the main responsible for disease diagnosis and patient monitoring, being involved in all the procedures performed to the patient (except for microperimetry, taken by the imaging technicians). Together with the retina specialist, the specialty trainee is responsible for slit lamp fundus examination, fluorescein angiography, indocyanine green angiography (ICGA), optical coherence tomography (OCT), angio-OCT, and fundus autofluorescence. According to the experience of the study participants, optometrists and nurses usually provide support with Snellen and/or ETDRS (Early Treatment Diabetic Retinopathy Study) tests even though they could, eventually, also provide support in other tasks such as OCT, fluorescein angiography, angio-OCT, or fundus autofluorescence.

**Figure 1:**
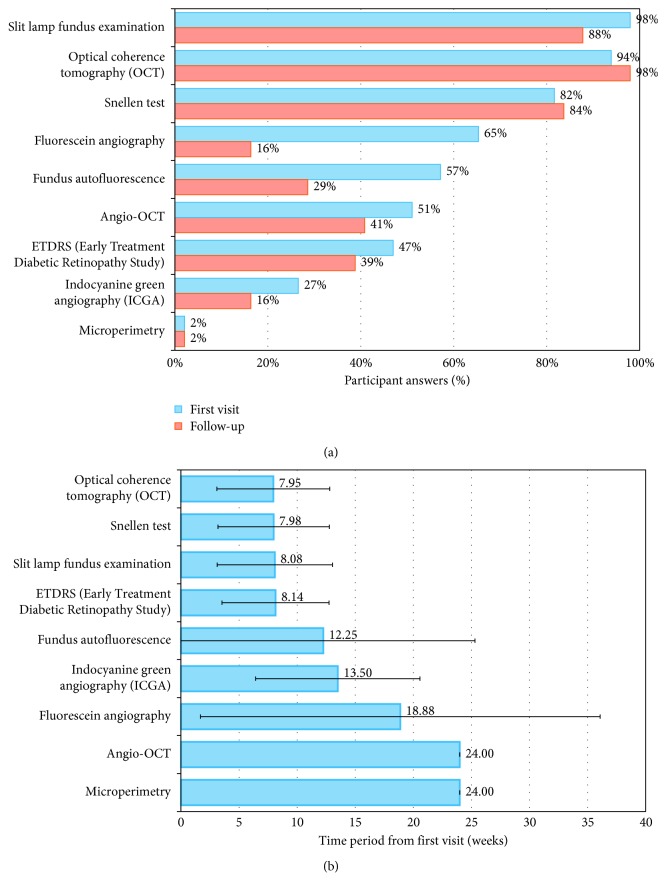
Use of diagnosis tests in routine clinical practice of the participant centres (*n* = 49), in patients with wAMD in Spain (multiple-choice question): (a) diagnosis tests; (b) time interval from wAMD diagnosis to first visit.

wAMD diagnosis is usually done by slit lamp fundus examination (98%), OCT (94%), Snellen test (82%), and/or fluorescein angiography (85%) (Figure 1(a)). Except for fluorescein angiography, these tests are frequently repeated during follow-up visits, to check disease progression and to assess treatment effectiveness, with a mean frequency of 2 months (Figure 1(b)).

### 3.3. Intravitreal Administration Procedure

Most of the panelists (63%) reported to have specific protocols for wAMD management, being mandatory in 10% of cases. National SERV (*Sociedad Española de Retina y Vitreo*) guidelines [8] were considered as key reference documents for 55% of the participants, while EURETINA guidelines [7] were mainly considered as supportive references for wAMD management (51% of the panelists).

Intravitreal administration by one-stop visit was possible in 57% of participant centres. Main limitations to conduct this practice were due to lack of staff (57%), short visit time (52%), limited access to the drug during the visit (52%), and facility issues (52%). Access to anti-VEFG drugs was not identified as issue, with the exception of sodium pegaptanib, restricted in 71% of the participant centres.

### 3.4. wAMD Treatment Pathways

#### 3.4.1. Loading Dose

With independence of the patient profile or lesion type, most of the panelists (92%) referred the use of a standard loading dose for anti-VEGF treatment initiation (three monthly injections), using as drug choice aflibercept in 40% of the patients, ranibizumab in 36%, and bevacizumab in 23%.

According to the experience of the panelists, 94% (SD 5%) of patients would complete appropriately the loading dose schedule.

#### 3.4.2. Maintenance Therapy

Maintenance therapy was done with the same anti-VEGF agent used during the loading phase period. During the first and second year, 81% of the patients would be treated with aflibercept and ranibizumab (first year: 43% and 38; second year: 45% and 36%, respectively). The remaining proportion of patients was treated with bevacizumab. No patients were treated with sodium pegaptanib.

**Figure 2:**
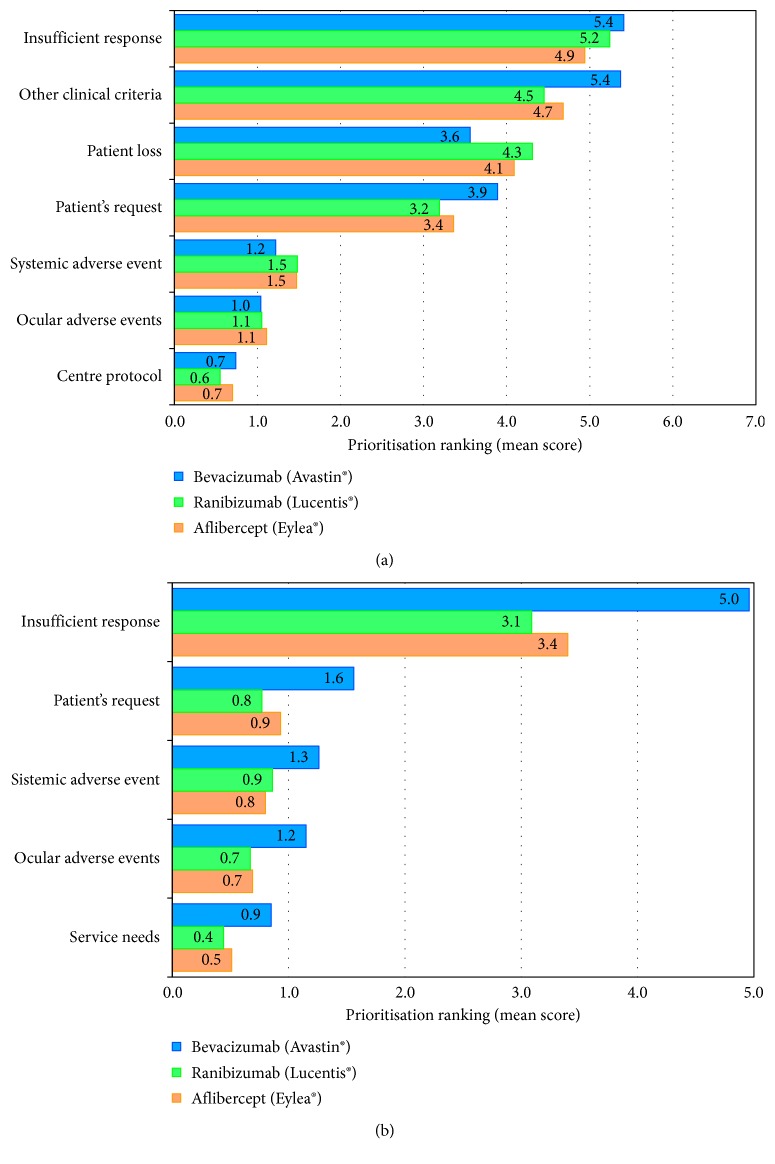
Prioritization made by the retina specialists regarding the main reasons for (a) anti-VEGF treatment dropout (7 points score) and (b) anti-VEGF switch (5 points score).

Overall, no major safety concerns were reported with anti-VEGF therapies, even when planned and used for a long time. According to the experience of the participants, 15% of the patients require to stop treatment (19% bevacizumab, 12% ranibizumab, and 10% aflibercept), mainly due to insufficient treatment response, other clinical criteria, and issues with patient follow-up (Figure 2(a)).

Moreover, around 23% of patients need to be switched to another agent (24% bevacizumab, 23% ranibizumab, and 15% aflibercept), usually because of an insufficient response to the initial treatment (Figure 2(b)). In these cases, the most frequent switch was to aflibercept or ranibizumab, depending on the first drug used.

#### 3.4.3. Treatment Dosing Strategy

**Figure 3:**
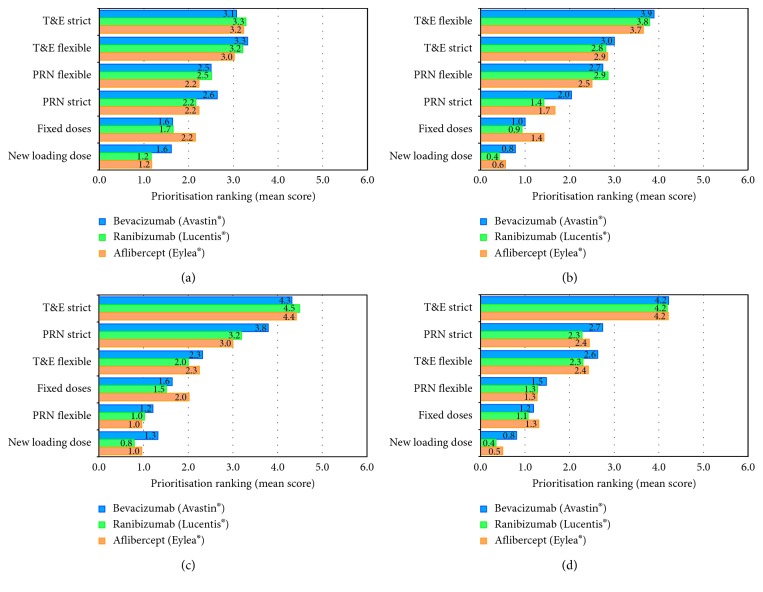
Treatment strategies prioritized by the retina experts (6 points score) (a) according to use during the first year of treatment, (b) according to use during the second year of treatment, (c) when considered as optimal for the first year of treatment, and (d) when considered as optimal for the second year of treatment.

The most frequently reported treatment regimens for wAMD patients were T&E and PRN (Figure 3). During the first year, the planned maintenance dosing strategies were strict T&E and PRN, whenever possible (Figure 3(a)). However, during the second year, these strategies needed to be adapted to a defined as “flexible” scheduling (Figure 3(b)).

According to the 39% and 37% of the panelists, the treatment strategies used during the first and second year, respectively, would not be considered as optimal. In the opinion of the 78% of participants, the most appropriate treatment strategy for the whole wAMD treatment should be strict T&E approach (Figures 3(c) and 3(d)), with independence of the patient profile or lesion type. The only exception would be identified in case of adverse events risk, where PRN would be considered the most appropriate approach (Table 1).

### 3.5. Challenges and Limitations for wAMD Management on Routine Clinical Practice

**Figure 4:**
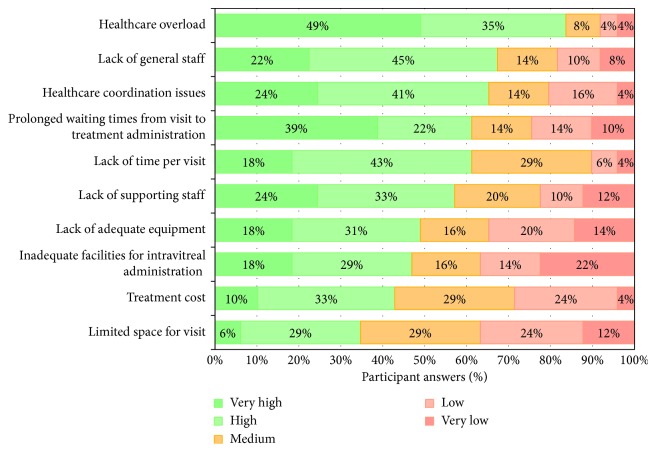
Impact of different healthcare factors on treatment choice, according to the routine clinical practice of the 49 retina specialists participating in the Delphi study.

**Figure 5:**
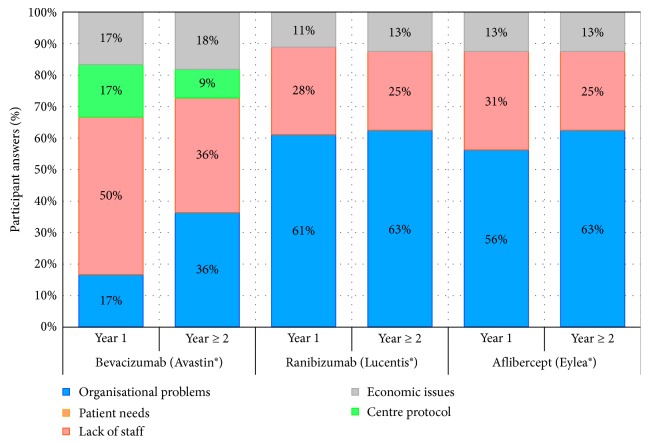
Main limitations for the establishment of the optimal therapeutic strategy in routine clinical practice, according to the opinion and experience of the 49 retina specialists participating in the Delphi study.

According to the experience of the study participants, treatment choice would be conditioned by healthcare overload (understood as lack of agenda for an appropriate patient monitoring according to the strict dosing strategy selected), lack of staff, healthcare coordination issues, and prolonged waiting time for intravitreal injection administration (Figure 4). In addition, the main limitations for an optimal treatment strategy achievement would be mainly related with organisational problems, confusion over scheduling, and healthcare resource availability (Figure 5).

## 4. Discussion

According to the main clinical guidelines and wAMD management protocols [7, 8], anti-VEGF therapies are the gold standard for wAMD treatment, and, actually, these are the therapies used on routine clinical practice in Spain. However, as it was evidenced in previous studies [13, 14], these good practices seem not to be reflected on outcomes, achieving suboptimal results compared with the expected by the anti-VEGF clinical trials [12, 20–22]. The main reason for these different results could be the difference in the management patterns among the strict injection protocols used in clinical trials and the adapted injection patterns used in routine clinical practice [16, 23–25] and identified as “flexible” in our study.

In general terms, according to the information provided by the study panelists, in Spain, wAMD management is mainly done according to the national and international retina guidelines (SERV and EURETINA) [7, 8] even though adapted to specific management protocols that could vary according to the healthcare resources and limitations of each centre and that could conduct to those “flexible” strategies.

A common fact among healthcare centres is that the main responsible for wAMD management is the retina specialist, involved from diagnosis to long-term monitoring, and including standard visits and associated procedures. Retina specialist tasks are supported by specialist trainees and, when available, by other healthcare staff such as nurse or optometrists. This complete involvement of the retina specialist in the patient management, although an advantage in terms of healthcare assistance quality for the patient, represents an important overload for the specialist, that finally conduct to a limitation for an optimal disease management and one-stop procedures.

In terms of treatment, the standard management of the patients is anti-VEGF therapies, according to the recommended practices [7, 8]. The main drugs used in routine clinical practice, considering all the treatment pathway, are aflibercept and ranibizumab, for both, loading dose and treatment maintenance, and being considered as the most appropriated for all patient's management, with independence of the patient profile or the type of lesion. According to the information provided by the study panelists, although no significant issues are evidenced for the loading dose (successful administration in more than 90% of the patients), important management issues for long-term schedules are identified, being mainly related with healthcare resource limitations, as well as their own healthcare overload and staff restrictions.

As it was anticipated in 2014 [16] and aligned with the available evidence [16, 24, 26, 27], most of the retina specialists participating in the study (78%) agreed in the aim for using T&E strategies in their routine clinical practice. However, healthcare reality and the limited resources available conduct to a need for self-defined “flexible” strategies that could be a critical issue for achieving optimal healthcare results in routine clinical practice.

Traditionally, reactive regimens for wAMD management were the standard procedures in Spain [8]. Strict PRN pattern was the reference protocol used in most centres even though not to be realistically conducted [12, 16]. Strict PRN protocols are linked to regular monthly visits, where disease monitoring is mandatory, and only in case of disease progression, the patient would be retreated, ideally in a one-stop visit [8]. Different studies have evidenced that this PRN model is difficult to achieve in routine clinical practice due to the limitations that the ophthalmology routine exercise have [12, 16]. Actually, with a quarter of the retina specialist workload focused on wAMD and a median of 15 minutes for patient care (including injection time), it is really difficult to achieve a strict PRN dosing strategy. In addition, considering that the most frequently used monitoring tests in Spain (slit lamp fundus examination, OCT, and Snellen test) would be conducted with a mean frequency of 8 weeks, it seems clear that the strict PRN protocol could not be appropriately conducted in routine clinical practice, as was confirmed by the panelists.

Recent studies indicate that a decrease in monitoring and injection frequency could not necessarily be related to negative impact on wAMD outcomes [24]. Then, strict PRN protocols could not be cornerstones for a successful wAMD management. Depending on disease progression and treatment response, the interval between injections could be progressively increased and even treatment could be completely stopped in case of disease stabilisation for more than 12 months [26]. In this regard, T&E strategies could be identified as optimal for wAMD management, in agreement with the opinion of our panelists.

T&E regimen could be defined as an individualized proactive dosing strategy, whose driver is the adaptation of the treatment regimen and visits interval according to disease activity, with the aim to achieve optimal outcomes [24, 26], similar to that provided with regular fixed dosing regimens [28, 29], but usually with a lower need for healthcare resources. However, the success of this strategy depends on the ability to perform the preplanned visits and effective treatment injection schedule, which is the main challenge in our current routine clinical practice and the main reason for defining this strategy as “flexible” for most of the panelists.

Delphi results evidenced many limitations that need to be addressed regarding wAMD management in Spain. In addition to the retina specialists overload and the lack of supporting staff that could be useful to optimise healthcare professional resources, the management of wAMD patients is affected by structural and organisational issues of the centres, making it difficult to schedule and conduct regular preplanned administration of intravitreal injections, one-stop management models, and appropriate treatment injection facilities, overall leading to long waiting periods from visit to injection administration (healthcare overload). Then, conducting to “flexible” management strategies for wAMD patients agreed as not effective enough.

Although these are the main issues identified in the Spanish context, they are not exclusive from Spain [25]. Other countries have also identified similar issues, agreeing in the need for investment in wAMD management [11, 30], with focus on better coordination among healthcare teams, workload reduction for retina specialists by increasing the involvement of optometrists or nurses in the management of wAMD patients, favouring one-stop model and the regular patient follow-up, with the aim of improving healthcare results.

Apart from the need for resources optimisation, treatment patterns could be supported by the use of drugs that favour timings adaptation and minimising the need for changing routine daily clinical practice and associated investment. In this regard, the use of drugs with a VEGF suppression longer than one month could have the potential to better suit to a proactive T&E regimen with a minor impact on resources [24]. These therapeutic options could potentially facilitate the appropriate implementation of strict T&E regimens that could improve treatment outcomes in routine clinical practice [27], achieving similar responses to those reported in the clinical trials [27, 29, 31] and agreeing with the opinion and experience of the study panelists regarding anti-VEGF choice.

The results of the present study showed that the healthcare reality in Spain has evolved from previous studies conducted in this country [12, 16], and it is similar to that observed in other countries despite the limitations of a Delphi study of these characteristics. Data provided in the study reflected the experience of 49 retina specialists representing their routine activity in the management of wAMD patients in Spain. All data were collected based on an accurate literature search and reviewed by a Steering Committee formed by reference Spanish retina specialists that confirmed results coherence according to their own experience and in agreement with data reported in the previous Delphi study performed in Spain [16], showing some improvement in the patient's management due to the adoption of proactive treatment strategies such as T&E and the authorisation of new therapeutic agents in the wAMD market. In addition, data were also confirmed by Delphi participants that validated the results provided in the first round by means of the answers of the second round.

Although data provided in this study should be confirmed by clinical charts and visit registries from healthcare centres, it seems clear that wAMD management is not optimal in most healthcare centres even though, in most cases, there exists the initiative to adopt proactive models for wAMD management. The use of agents allowing longer treatment intervals could help, but the results of this study indicate that additional organisational changes and resources reallocation beyond financial investment could be necessary, in order to provide a real improvement in the management of wet AMD patients, favouring the effective implementation of a T&E strategy.

## 5. Conclusions

Anti-VEGF administration is the cornerstone for wAMD treatment, based on a loading dose followed by a long-term treatment until disease stabilization. Traditionally, reactive treatment regimens were the standard procedure for wAMD management, usually focused on PRN dosing strategies. However, PRN regimen was linked to strict management protocols difficult to address in routine clinical practice, where healthcare resources are limited. Organisational healthcare limitations together with staff workload are the main reasons why neither monthly visit nor one-stop visits for immediate retreatment could be conducted. Then, evolution to proactive treatment models (T&E) became a priority in this situation, especially considering the potential advantages in terms of appointment scheduling, healthcare resource management, and increasing the interval period between visits.

In this regard, as healthcare outcomes are linked to a regular administration of the intravitreal therapy, mostly on a proactive regimen basis, such as T&E, the use of drugs allowing long administration intervals could contribute to the wAMD management optimisation in routine clinical practice. In addition, optimal and preplanned drug administration frequencies could contribute to an improvement of the workload for retina specialists and the possibility for a regular one-stop administration of these therapies with a minimum impact on the centre's resources.

## Figures and Tables

**Table 1:** Panelists opinion (*n* = 49) regarding anti-VEGF treatment strategies considered as optimal for wAMD management according to patient profile and lesion type in Spain.

	PRN strict	PRN flexible	Fixed dose	T&E strict	T&E flexible	No treatment
*Patient characteristics; opinion, n (%)*						
Very low initial VA	12 (24%)	6 (12%)	3 (6%)	25 (51%)	7 (14%)	6 (12%)
Good initial VA	10 (20%)	1 (2%)	9 (18%)	34 (69%)	3 (6%)	0 (0%)
Aged patient	7 (14%)	2 (4%)	9 (18%)	24 (49%)	13 (27%)	1 (2%)
Single eye	14 (29%)	0 (0%)	11 (22%)	34 (69%)	1 (2%)	0 (0%)
Adverse events risk	16 (33%)	9 (18%)	1 (2%)	12 (24%)	10 (20%)	2 (4%)
Centre accessibility (distance)	4 (8%)	4 (8%)	7 (14%)	26 (53%)	10 (20%)	0 (0%)
Protocol/centre guidelines	12 (24%)	3 (6%)	8 (16%)	27 (55%)	10 (20%)	0 (0%)
Patient cost	13 (27%)	10 (20%)	4 (8%)	15 (31%)	11 (22%)	1 (2%)

*Disease characteristics (type of lesion); opinion, n (%)*						
Neovascularisation type 1	10 (20%)	0 (0%)	8 (16%)	37 (76%)	5 (10%)	0 (0%)
Neovascularisation type 2	13 (27%)	0 (0%)	10 (20%)	37 (76%)	2 (4%)	0 (0%)
Neovascularisation type 3	15 (31%)	0 (0%)	13 (27%)	33 (67%)	3 (6%)	0 (0%)

VA: visual acuity.

## Data Availability

According to Delphi methodology, the study data were collected from panelists' answers in a specific study database. This database would not be publicly available even though ad hoc data or analysis could be provided upon request to the corresponding author.
